# Beiträge zur Ösophagus-Chirurgie

**Published:** 1913-12

**Authors:** 


**Beiträge zur Ösophagus-Chirurgie.** Von Dr. Alwin Ach. iv, 136, S. München: J. F. Lehmann's Verlag. 1913. Preis M.4.

In this book the reader will find descriptions of most of the methods which have been employed by the author and other surgeons in resection of the œsophagus. More particularly the author describes his own operations of the kind, carried out on fifty-five dogs and five human beings, the latter having been patients suffering from carcinoma of the œsophagus. The results of the operations were not brilliant, the best being, we found, that a dog survived over three months and a man survived seventeen days. It is clear, therefore, that the difficulties and dangers of removing a portion or the whole of the intrathoracic part of the œsophagus are immense. Still advances are being made, and some of the lines taken in the progress are well indicated in this work. For example, the author has evolved and proved the practicability of removing the upper segment of a divided intra-thoracic œsophagus by invagination from below upwards. To effect this an instrument is passed down the œsophagus from the mouth to the divided and ligatured end, attached there, and used for traction. Subsequently an evagination is made through an incision in the neck, and the œsophagus thus transplanted externally for œsophagoplasty.

Though the writer's results, like those of other pioneers in this branch of surgery, leave so much to be desired, yet they inspire hope for the future, and the records of his investigations and experiences here given demand the serious consideration of all surgeons who contemplate attempting resection of the œsophagus.

